# Sensorimotor network dynamics predict decline in upper and lower limb function in people with multiple sclerosis

**DOI:** 10.1177/13524585221125372

**Published:** 2022-09-30

**Authors:** Myrte Strik, Anand JC Eijlers, Iris Dekker, Tommy AA Broeders, Linda Douw, Joep Killestein, Scott C Kolbe, Jeroen JG Geurts, Menno M Schoonheim

**Affiliations:** Melbourne Brain Centre Imaging Unit, Department of Radiology, University of Melbourne, Melbourne, VIC, Australia/MS Center Amsterdam, Anatomy and Neurosciences, Vrije Universiteit Amsterdam, Amsterdam Neuroscience, Amsterdam UMC location VUmc, Amsterdam, the Netherlands; MS Center Amsterdam, Anatomy and Neurosciences, Vrije Universiteit Amsterdam, Amsterdam Neuroscience, Amsterdam UMC location VUmc, Amsterdam, the Netherlands; MS Center Amsterdam, Neurology, Vrije Universiteit Amsterdam, Amsterdam Neuroscience, Amsterdam UMC location VUmc, Amsterdam, the Netherlands; MS Center Amsterdam, Anatomy and Neurosciences, Vrije Universiteit Amsterdam, Amsterdam Neuroscience, Amsterdam UMC location VUmc, Amsterdam, the Netherlands; MS Center Amsterdam, Anatomy and Neurosciences, Vrije Universiteit Amsterdam, Amsterdam Neuroscience, Amsterdam UMC location VUmc, Amsterdam, the Netherlands; MS Center Amsterdam, Neurology, Vrije Universiteit Amsterdam, Amsterdam Neuroscience, Amsterdam UMC location VUmc, Amsterdam, the Netherlands; Department of Neurosciences, Central Clinical School, Monash University, Melbourne, VIC, Australia; MS Center Amsterdam, Anatomy and Neurosciences, Vrije Universiteit Amsterdam, Amsterdam Neuroscience, Amsterdam UMC location VUmc, Amsterdam, the Netherlands; MS Center Amsterdam, Anatomy and Neurosciences, Vrije Universiteit Amsterdam, Amsterdam Neuroscience, Amsterdam UMC location VUmc, Amsterdam, the Netherlands

**Keywords:** Functional magnetic resonance imaging, upper and lower limbs, disability progression, network efficiency, network dynamics, longitudinal

## Abstract

**Background:**

Upper and lower limb disabilities are hypothesized to have partially independent underlying (network) disturbances in multiple sclerosis (MS).

**Objective:**

This study investigated functional network predictors and longitudinal network changes related to upper and lower limb progression in MS.

**Methods:**

Two-hundred fourteen MS patients and 58 controls underwent functional magnetic resonance imaging (fMRI), dexterity (9-Hole Peg Test) and mobility (Timed 25-Foot Walk) measurements (baseline and 5 years). Patients were stratified into progressors (>20% decline) or non-progressors. Functional network efficiency was calculated using static (over entire scan) and dynamic (fluctuations during scan) approaches. Baseline measurements were used to predict progression; significant predictors were explored over time.

**Results:**

In both limbs, progression was related to supplementary motor area and caudate efficiency (dynamic and static, respectively). Upper limb progression showed additional specific predictors; cortical grey matter volume, putamen static efficiency and posterior associative sensory (PAS) cortex, putamen, primary somatosensory cortex and thalamus dynamic efficiency. Additional lower limb predictors included motor network grey matter volume, caudate (dynamic) and PAS (static). Only the caudate showed a decline in efficiency over time in one group (non-progressors).

**Conclusion:**

Disability progression can be predicted using sensorimotor network measures. Upper and lower limb progression showed unique predictors, possibly indicating different network disturbances underlying these types of progression in MS.

## Introduction

Loss of upper and lower limb functions are two of the most common and disabling symptoms of multiple sclerosis (MS), an inflammatory neurodegenerative disease of the central nervous system.^1,2^ The lower limbs are often affected early in the disease and more severely than the upper limbs,^1,3^ yet more than 50% of patients with MS will also experience upper limb difficulties including tremor, clumsiness and muscle weakness.^2^ Interestingly, the severity of upper and lower limb impairments in individual patients correlate only modestly, suggesting at least partially different underlying processes^2^ and highlighting the need for personalized monitoring strategies.

Due to the known importance of the sensorimotor network for normal motor function, disability in MS can be appreciated in terms of both structural and functional damage to this network. Previous cross-sectional studies have indicated that functional network measures may provide additional insights beyond common radiological assessments of lesions load or atrophy.^4,5^ Such studies have shown complex patterns of connectivity in MS, including higher^4,5^ and lower^6^ sensorimotor connectivity. However, it remains unclear whether such functional changes also impact overall efficiency of the motor system and whether these findings are predictive, as longitudinal studies are rare.^7,8^ In addition to longitudinal work, studies investigating network correlates of upper and lower limb dysfunction specifically are also lacking, which leaves underlying mechanisms unclear.

In this study, we therefore aimed to investigate how longitudinal changes in upper and lower limb disability are related to functional changes to the sensorimotor network, which we hypothesized would show specific patterns depending on the type of impairment. To investigate our hypothesis, we assessed sensorimotor network ‘efficiency’,^9^ a graph theoretic measure of a network node’s connectedness to other nodes that has previously been applied to the study of cognitive dysfunction^10^ and overall disability^7^ in MS. In addition to static measurements across the entire resting-state functional magnetic resonance imaging (fMRI) scan, we also used a dynamic approach, which was recently validated in previous studies.^11,12^ With dynamics, the temporal fluctuations during a scan are calculated and can be used as a measure of network stability.

## Methods

### Participants

This paper assessed longitudinal retrospective data from the prospectively acquired Amsterdam MS cohort,^11^ including only those subjects who attended both baseline (2008–2012) and follow-up (FU) (2014–2017) assessments after 5 years, resulting in 234 patients (70%, 234/332) and 60 controls (63%, 60/96). All patients were diagnosed with clinically definite MS according to the 2010 revised McDonald criteria.^13^ Exclusion criteria included MS relapse in 2 months prior to MRI and history or presence of psychiatric and/or neurological disease other than MS.^14^ Disease-modifying treatments (DMTs) included glatiramer acetate (*n* = 12), natalizumab (*n* = 12), β-interferons (*n* = 56) or other immunosuppressive therapy (*n* = 4), and 140 patients were not on any DMT. This study focused specifically on motor impairments so patients were included if they: (a) had whole-brain and cerebellar resting-state fMRI coverage and (b) upper and/or lower limb (able to walk) functional tests at both time points. This resulted in a final sample of 214 patients (47 ± 11 years; 149 women) and 58 healthy controls (46 ± 10 years; 31 women).

### Upper and lower limb disability assessments and classification of patients

Overall, disability was assessed using the Expanded Disability Status Scale (EDSS).^15^ Upper limb disability (i.e. dexterity) was assessed using the 9-Hole Peg Test.^16^ Performance of dominant and non-dominant hands was averaged. Lower limb disability (i.e. mobility) was assessed using the Timed 25-Foot Walk Test^17^ with the averaged time of two walks. Upper and lower limb disability progression was defined as at least a 20% decline in performance on the respective tests, as previously reported.^18^

### Magnetic resonance imaging acquisition

Imaging was performed on a 3-Tesla GE magnetic resonance imaging (MRI) system (Signa HDxt, Milwaukee, WI, USA) with an eight-channel phased-array head coil. A hardware upgrade (gradient system among others) took place between scan sessions, which was corrected for using a procedure previously published using the same data set, based on *Z*-score transformations (based on healthy control mean value and standard deviations) at each time point.^14^ The impact of (re)positioning on outcomes was assessed using standardized pipelines. The image acquisition protocol included resting-state fMRI, acquired with an echo-planar imaging (EPI) sequence covering whole-brain repetition time (TR) = 2200 ms, time to echo (TE) = 35 ms, flip angle (FA) = 80°, 3 mm contiguous axial slices, in-plane 3.3 × 3.3 mm^2^, 202 volumes. For brain volumetric measurements, a 3DT1 FSPGR was used (TR = 7.8 ms, TE = 3.0 ms, FA = 12°, inversion time (TI) = 450 ms, 1.0 mm sagittal slices, 0.9 × 0.9 mm^2^ in-plane resolution) and fluid-attenuated inversion recovery (FLAIR) for lesion detection (TR = 8000 ms, TE = 125 ms, TI = 2350 ms, 1.2 mm sagittal slices, 0.98 × 0.98 mm^2^ in-plane resolution).

### Brain measures: white matter lesion and brain volumes

Lesions were semi-automatically detected and segmented on FLAIR^19^ and filled on three-dimensional T1 scans using Lesion Automated Pre-processing (LEAP). Total brain, white matter (WM), cortical and deep grey matter (GM) volumes were calculated using SIENAX and FIRST (FSL5, Oxford, UK; https://fsl.fmrib.ox.ac.uk/fsl/). The sensorimotor network GM volume was calculated using the Brainnetome atlas (http://atlas.brainnetome.org).^20^ Volumes were corrected for head size using V-scaling from SIENAX.

### Resting-state fMRI pre-processing

Resting-state fMRI data was pre-processed using MELODIC (FSL5, FMRIB 2012, Oxford, UK; https://fsl.fmrib.ox.ac.uk/fsl/, standard settings, spatial smoothing 5 mm). Further pre-processing involved WM and cerebrospinal fluid signal regression, images were checked for artefacts and registration errors and further motion regression using ICA-AROMA (v0.4-beta 2017, Nijmegen, the Netherlands).^21^ Subsequently, voxels without reliable signal (EPI-distortions artefacts or non-brain tissue) were excluded using a robust range–based threshold and high-pass temporal filtering (100 seconds cut-off), as previously reported.^11^ Registration parameters between fMRI and lesion-filled 3DT1 were calculated with boundary-based registration and the standard brain using FNIRT (FSL5), both of which were inverted (FSL5).

### Regions of interest

The Brainnetome atlas was registered to each individual T1-weighted scan and multiplied with individual GM masks derived from SIENAX analysis (FSL5). A cerebellar region of interest (ROI) was created from the Harvard–Oxford atlas (FSL5), registered to each individual T1-weighted scan and deep GM structures were segmented on T1-weighted scans using FIRST (FSL5). Cortical and subcortical ROIs were combined, registered to each individual fMRI scan and assessed on sufficient reliable signal, that is, each ROI required at least 30% voxels remaining in at least 90% of subjects after removing unreliable voxels. This approach led to removal of inferior temporal, orbitofrontal and nucleus accumbens areas, resulting in 193 ROIs. The sensorimotor system was defined based on an approach used previously,^22,23^ including 23 cortical (frontal, primary and secondary motor and sensory ROIs) and subcortical (cerebellum, thalamus, caudate nucleus, putamen and pallidum) ROIs.

### Efficiency of the sensorimotor system

**Figure 1.**
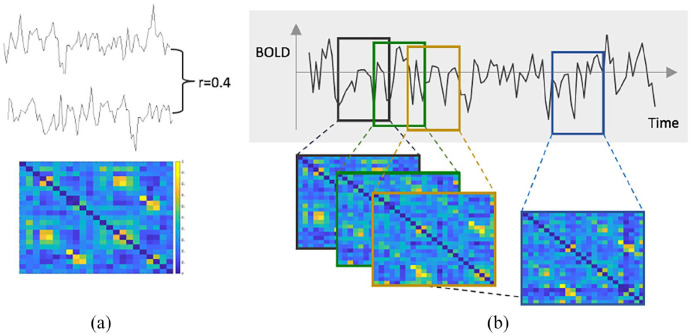
Dynamic connectivity and efficiency of the sensorimotor network. (a) Matrix of the sensorimotor network connectivity during one window of a single subject. The colour bar reflects Pearson’s correlation, that is, the connectivity strength. (b) To compute the dynamic efficiency, a shifting window approach was used (shift of five volumes), resulting in partially overlapping windows. For each window, the connectivity was calculated and network efficiency was computed. To quantify the dynamics, the relative standard deviation over all windows for each sensorimotor region was calculated. L: Left; R: Right; PreF: prefrontal cortex; SMA: supplementary motor area; Premot: premotor cortex; M1: primary motor cortex; S1: primary somatosensory cortex; S2: primary sensory cortex; Post assoc: posterior associative sensory cortex; Tha: thalamus; Cau: caudate nucleus; Put: putamen; Pal: pallidum.

For each subject, signal intensities were averaged for each volume within each individual ROI to form 193 time series. All subsequent analyses were performed in MATLAB (MathWorks, R2018b, Natick, MA, USA) using custom scripts. Stationary functional connectivity (FC) was calculated using correlations between all ROIs. The sensorimotor system was extracted (23 × 23 submatrix), and each connection was corrected for whole-brain connectivity, to reduce inter-individual differences. To assess dynamic FC, partially overlapping sliding windows of 27 time points (59.4 seconds) and a shift length of five volumes (11 seconds) were applied, resulting in 34 windows per scan (Figure 1), comparable to previously suggested settings.^24^ For each window, FC was calculated (23 × 23 × 34 matrix), and dynamic FC was defined as the coefficient of variation over time for each connection. For both dynamic and static connectivities, absolute Pearson’s correlation values were used.

Next, we studied the most basal and crucial aspects of a network, integration and segregation.^25^ To assess information propagation in the brain at a global (segregation) and local (integration) level, network efficiency was calculated on the static connectivity matrix using the Brain Connectivity Toolbox (https://sites.google.com/site/bctnet/). Global efficiency is inversely related to average shortest path length, that is, how many steps are required to connect regions. Local efficiency is related to clustering coefficient, that is, a network measure of the degree to which neighbour nodes cluster together.^26^ To derive dynamic network measures, this process was repeated for each window, and the coefficient of variation was calculated, as described above.

### Null models

Dynamic FC and efficiency measures were compared with null-models to assess their deviation from random noise. These surrogate data were acquired using phase-randomization of the data after the Fourier transformation, averaging connectivity and efficiency measures across 50 randomization runs for each subject.^27^ All dynamic functional efficiency measures were significantly different compared to measures calculated using null-models, indicating non-random efficiency fluctuations (all *p* < 0.001).

### Statistical analysis

Statistical analyses were performed using SPSS 26 (IBM, Chicago, IL, USA). Variables were checked for normality using the Kolmogorov–Smirnov test and visual inspection of histograms, and compared between groups using generalized linear model (GLM). Efficiency variables were converted into *Z*-scores; lesion volumes were log-transformed. Non-normally distributed variables were analysed with the Mann–Whitney *U* tests.

To identify the main correlates of disability progression over 5 years’ time, three initial unimodal binary logistic regression with backward selection were performed including either (1) baseline structural measures (brain volumes and lesion loads), (2) baseline dynamic or (3) baseline static sensorimotor MRI measures. Significant baseline measures were included in a binary logistic regression model (backward selection) to identify the final selection of cross-modal correlates of progression. Only significant functional MRI predictors were then evaluated over time, to limit the number of comparisons, using repeated GLM and post hoc paired *t*-tests. For longitudinal assessment of structural MRI measures,^14^ sex, age and time interval between scan sessions were included as covariates. *p*-values < 0.05 were considered statistically significant, and analyses were Bonferroni-corrected over number of tests.

## Results

### Demographic and clinical characteristics

**Figure 2.**
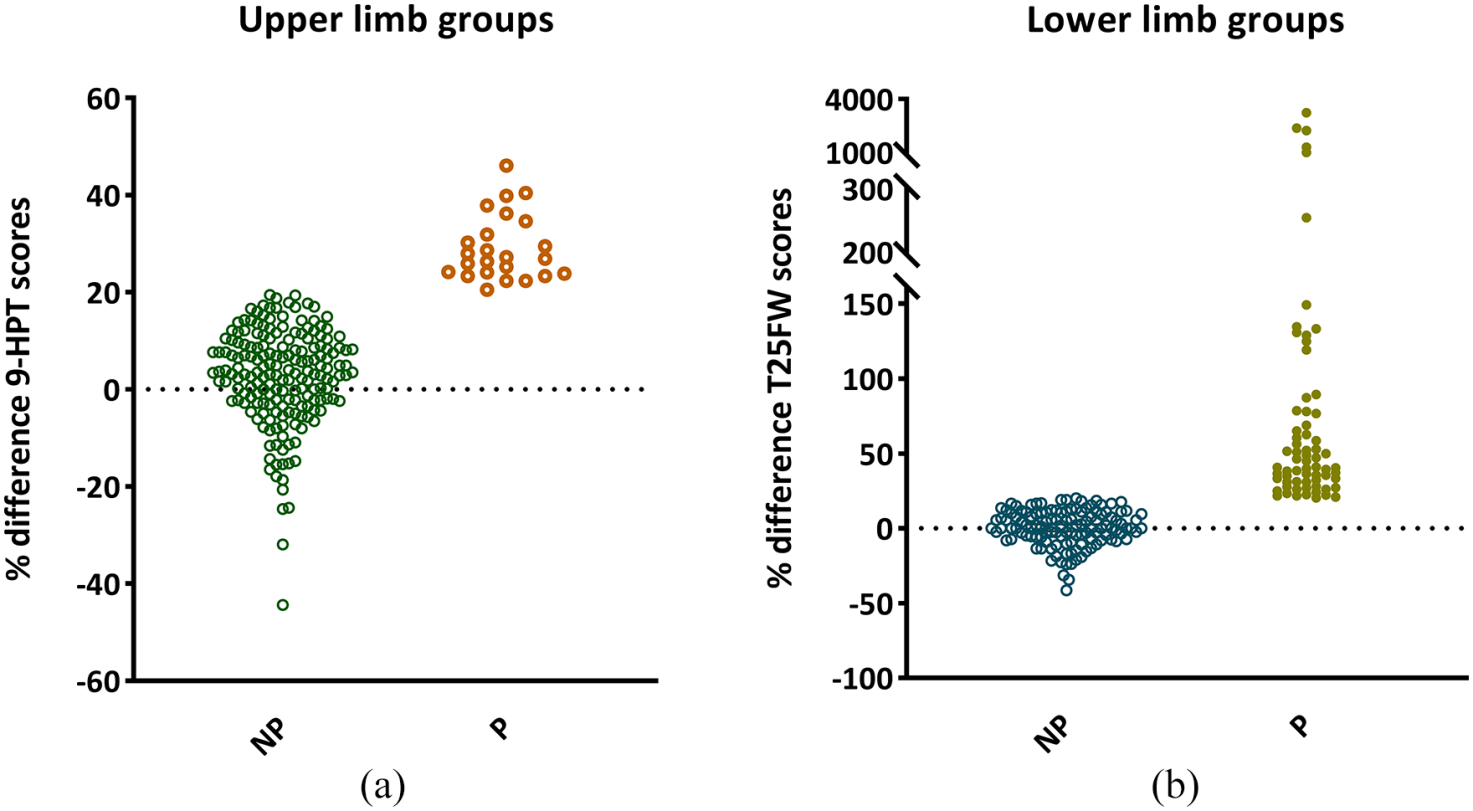
Percentage change in upper and lower limb performance over time. Percentage change in performance scores on the 9-Hole Peg (9-HPT) (a) and Timed 25 Foot Walk (T25FW) (b) tests, quantitative tests for upper and lower limb function, respectively. (a) One-hundred eighty-six patients were categorized as upper limb non-progressing (NP) patients and 24 patients were defined as upper limb progressing (P). Of the NP patients, 62 out of 186 (33%) scored lower at follow-up, which means a better performance, with the remainder scoring <20% worse. (b) Sixty-seven patients were defined as lower limb P and 124 as NP patients of which 52 patients (42%) improved, all remainder patients performed slightly worse over time (<20%).

Baseline demographics, clinical characteristics and brain volumes are shown in Table 1. Median EDSS progressed over time (from 3.0 to 3.5, *p* < 0.001) and 21/172 (12%) patients converted to secondary progressive MS. Patients were divided (Figure 2) into upper limb non-progressing (*n* = 186, age 47 ± 11 years, 35.5% DMT) and progressing (*n* = 24, age 49 ± 13 years, 45.8% DMT) and lower limb non-progressing (*n* = 124, age 47 ± 11 years, 37.1% DMT) and progressing (*n* = 67, age 46 ± 11 years, 43.3% DMT). Thirteen patients progressed on both tests. No differences were observed between the number of right and left handed patients in both upper (*p* *=* 0.924) and lower (*p* = 0.509) limb groups. Lower limb progressors had a higher EDSS (*p* = 0.001) at FU and were more commonly man (43.3% (29/67) vs 24.2% (30/124), *p* = 0.007) compared to non-progressing patients. No differences were seen between upper limb progressing and non-progressing patients on demographics nor on baseline upper and lower limb functioning.

**Table 1.** Demographics, clinical variables and MRI characteristics.

	Upper limb non-progressing (*n* = 186)	Upper limb progressing (*n* = 24)	Lower limb non-progressing (*n* = 124)	Lower limb progressing (*n* = 67)	Healthy controls (*n* = 58)
**Demographics**					
Sex, W/M	128/58	18/6	94/30	38/29**	31/27
B age, years	47 (11)	49 (13)	47 (11)	46 (11)	46 (10)
B symptom duration, years	15 (9)	14 (7)	15 (8)	13 (7)	
Time interval B and FU, years	5 (1)	5 (1)**	5 (1)	5 (1)	6 (1)
B treatment (yes/no)	66/120	11/13	46/78	29/38	
B phenotypes (RR/SP/PP), *n*	150/26/10	20/2/2	109/12/3	53/8/6	
RRMS converted to SPMS at FU, *n*	15 (10%)	6 (30%)	14 (12.8)	6 (11.3%)	
B EDSS^a^	3.0 (8.0)	3.5 (5.5)	3.0 (6.5)	3.0 (6.5)	
FU EDSS^a^	3.0 (8.5)	4.0 (6.0)	3.0 (6.0)	4.0 (7.5)**	
Dominant hand (right/left), *n*	164/22	21/3	109/15	61/6	
B can walk (yes/no/missing), *n*	166/3/17	23/0/1	112/0/12	62/0/5	
FU can walk (yes/no/missing), *n*	167/5/14	20/0/4	116/0/8	57/1/9	
B 9-HPT, seconds^a^	19.4 (155.3)	19.2 (23.3)	19.25 (23.3)	19.33 (146.1)	
FU 9-HPT, seconds^a^	20.03 (80)	24.95 (29.93)	19.95 (32.8)	21.13 (30.75)	
B T25FW, seconds^a^	4.3 (177.7)	4.25 (21.9)	4.3 (12.3)	4.1 (13.8)	
FU T25FW, seconds^a^	4.8 (177.1)	5.35 (176.5)	4.4 (12.5)	5.94 (177.1)	
Brain volumes					
NWMV, L	0.67 (0.03)*	0.67 (0.05)*	0.67 (0.03)*	0.68 (0.04)*	0.70 (0.03)
NCGMV, L	0.76 (0.05)*	0.72 (0.05)*,**	0.76 (0.05)*	0.75 (0.05)*	0.78 (0.05)
NDGMV, L	0.06 (0.01)*	0.06 (0.01)*	0.06 (0.01)*	0.06 (0.01)*	0.06 (0.00)
NMNGMV, L	0.47 (0.05)*	0.49 (0.07)	0.47 (0.05)*	0.48 (0.06)	0.49 (0.03)
Lesion volume (log), mL	3.93 (0.39)	4.1 (0.33)	3.93 (0.38)	3.94 (0.4)	

W: women; M: men; B: baseline; FU: follow-up; RR: relapsing-remitting; SP: secondary progressive; PP: primary progressive; EDSS: Expanded Disability Status Scale; 9-HPT: 9-Hole Peg Test; T25FW: Timed 25-Foot Walk test; RRMS: relapsing-remitting multiple sclerosis; SPMS: secondary progressive multiple sclerosis; NWMV: normalized white matter volume; NCGMV: normalized cortical grey matter volume; NDGMV: normalized deep grey matter volume; NMNGMV: normalized motor network grey matter volume.

All values represent mean values and standard deviations unless denoted otherwise.

a Median and range.

Significant difference compared to healthy controls (*) and non-progressing patients (**).

### Brain and lesion volumes in progressing and non-progressing patients

At baseline, compared to controls, all four MS groups showed significantly lower WM, cortical and deep GM volumes (Table 1, *p* < 0.05). Compared to controls, patients with higher disability levels (EDSS >4) showed significant atrophy compared to controls (*p* = 0.007), while less disabled patients did not. In addition, non-progressing upper (*p* = 0.010) and lower (*p* = 0.003) limb patients showed lower motor network volume (i.e. patients with atrophy had worse disability at baseline but were less likely to progress further). Patients with upper limb progression compared to non-progressing patients displayed a lower cortical grey matter (CGM) volume at baseline (*p* = 0.001), while no differences were found between lower limb groups for any volumetric measures. Structural progression in the same cohort has been described in a previous publication, demonstrating accelerated cortical atrophy in progressive MS in relation to cognitive decline.^14^

### Predictors of upper and lower disability progression in MS: three initial unimodal models

Brain volumes: the volumetric prediction model to identify the main correlates of progression included CGM, motor network GM volume and time interval for upper limb progression (Nagelkerke *R*^2^ = 0.22, chi-square = 25.14, *p* < 0.001) and CGM, motor network GM volume and male sex for lower limb progression (Nagelkerke *R*^2^ = 0.11, chi-square = 15.79, *p* = 0.001).
Static network efficiency: the static network efficiency prediction model for upper limb progression identified the predictive value of higher LE of the cerebellum, left secondary sensory cortex (S2), right premotor cortex, primary motor cortex (M1) and putamen as well as lower GE, left premotor cortex and right caudate, age and time interval (Nagelkerke *R*^2^ = 0.30, chi-square = 35.09, *p* < 0.001). For lower limb progression, the model identified higher LE of the cerebellum, left posterior associative sensory (PAS) cortex and right M1 and lower LE of the right thalamus and caudate and male sex (Nagelkerke *R*^2^ = 0.19, chi-square = 27.51, *p* < 0.001).
Dynamic network efficiency: the baseline dynamic network efficiency model predicting upper limb progression identified higher LE of the right supplementary motor area (SMA), premotor, primary somatosensory cortex (S1) and thalamus and lower LE of the left S2, PAS and putamen, age and time interval (Nagelkerke *R*^2^ = 0.37, chi-square = 43.29, *p* < 0.001). The model for lower limb progression identified higher right caudate and lower SMA dynamic efficiency and male sex (Nagelkerke *R*^2^ = 0.12, chi-square = 17.77, *p* < 0.001).

### Most important predictors of progression: multivariate model

Final predictors of upper and lower limb progression are described in Table 2.

**Table 2.** Significant predictors of upper and lower limb progression.

		Model				Predictor		
		Nagelkerke *R*^2^	Chi-square	*p*	*B*	SE	Wald	*p*
**UL progression predictors**		0.441	53.31	<0.001				
	Left PAS LE dynamics				−2.10	0.63	11.02	0.001
	Left putamen LE dynamics				−1.76	0.62	7.98	0.005
	Right SMA LE dynamics				1.11	0.59	3.51	0.061
	Right S1 LE dynamics				1.46	0.67	4.81	0.028
	Right thalamus LE dynamics				1.39	0.50	7.69	0.006
	Right caudate nucleus static LE				−1.18	0.44	7.24	0.007
	Right putamen static LE				0.93	0.32	8.53	0.003
	Time interval				0.86	0.35	6.06	0.014
	NCGM volume				−22.76	6.40	12.65	<0.001
**LL progression predictors**		0.257	39.40	<0.001				
	Right SMA LE dynamics				0.84	0.28	8.79	0.003
	Right caudate nucleus LE dynamics				−0.63	0.27	5.42	0.020
	Left PAS static LE				0.41	0.18	5.17	0.023
	Right M1 static LE*				0.41	0.19	4.62	0.032
	Right thalamus static LE*				−0.43	0.24	3.19	0.074
	Right caudate nucleus static LE				−0.67	0.25	7.22	0.007
	NMNGM volume				7.70	3.55	4.71	0.030
	Sex				1.75	0.43	16.77	<0.001

*B*: predictor specific *b*-value; SE: standard error; LE: local efficiency; LL: lower limb; M1: primary motor cortex; NCGM: normalized cortical grey matter volume; NMNGM: normalized motor network grey matter; PAS: posterior associative sensory cortex; S1: primary somatosensory cortex; SMA: supplementary motor area; UL: upper limb.

Logistic regression analyses with backward elimination were performed to find most important correlates of upper and lower limb progression. Predictors were considered statistically significant when *p* < 0.050.

* Not included in final prediction model after removal outliers (Figure 2).

Upper limb disability progression was related (Nagelkerke *R*^2^ = 0.44, chi-square = 53.31, *p* < 0.001) to lower static LE of the right caudate and dynamic efficiency of the left PAS and putamen and higher static efficiency of the right putamen and dynamic efficiency of the right SMA, S1 and thalamus, lower CGM volume and longer time interval.

Lower limb disability progression was related (Nagelkerke *R*^2^ = 0.26, chi-square = 39.40, *p* < 0.001) to higher static LE of the left PAS and right M1 and dynamic efficiency of the right SMA and lower static efficiency of the right thalamus and caudate nucleus and dynamic efficiency of the right caudate, higher motor network GM volume and male sex.

Repeating these regression models including hand dominance did not change these results. After visualizing longitudinal changes (Figure 2), five patients were identified who demonstrated a large difference between baseline and FU Timed 25-Foot Walk (T25FW) scores. To assess the influence of these outliers on the results, the lower limb multivariate analysis was repeated excluding these participants. This model showed similar results, although M1 and thalamus were no longer predictive and not further explored.

### Longitudinal network changes

**Figure 3.**
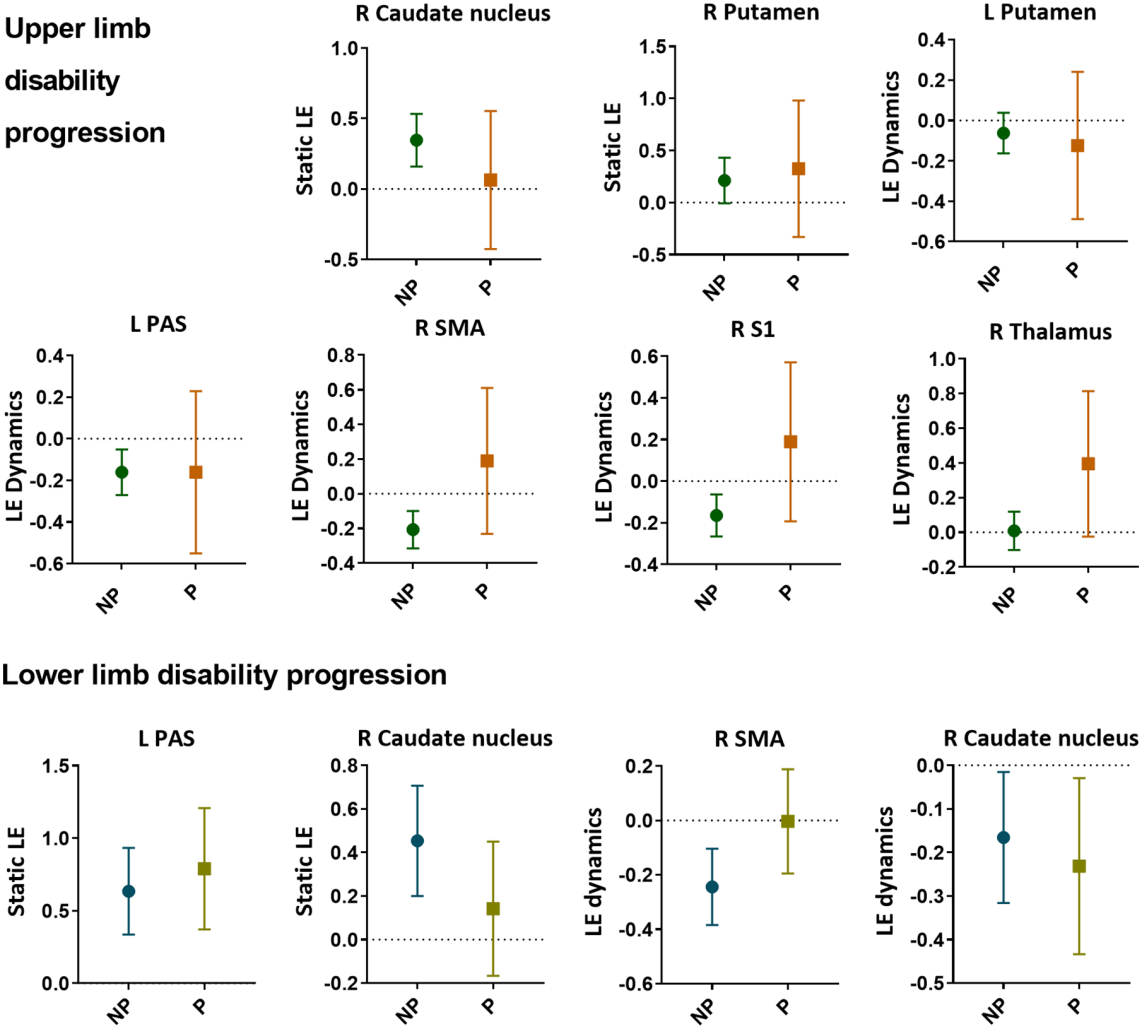
Baseline efficiency of significant correlates of upper and lower limb disability progression. Static (i.e. over the entire scan) as well as dynamic (i.e. fluctuations over time) network efficiency of several cortical and subcortical regions were predictive of decline in upper and/or lower limb function. The round and square symbols reflect the mean, and the error bar represents the 95% confidence interval.

**Figure 4.**
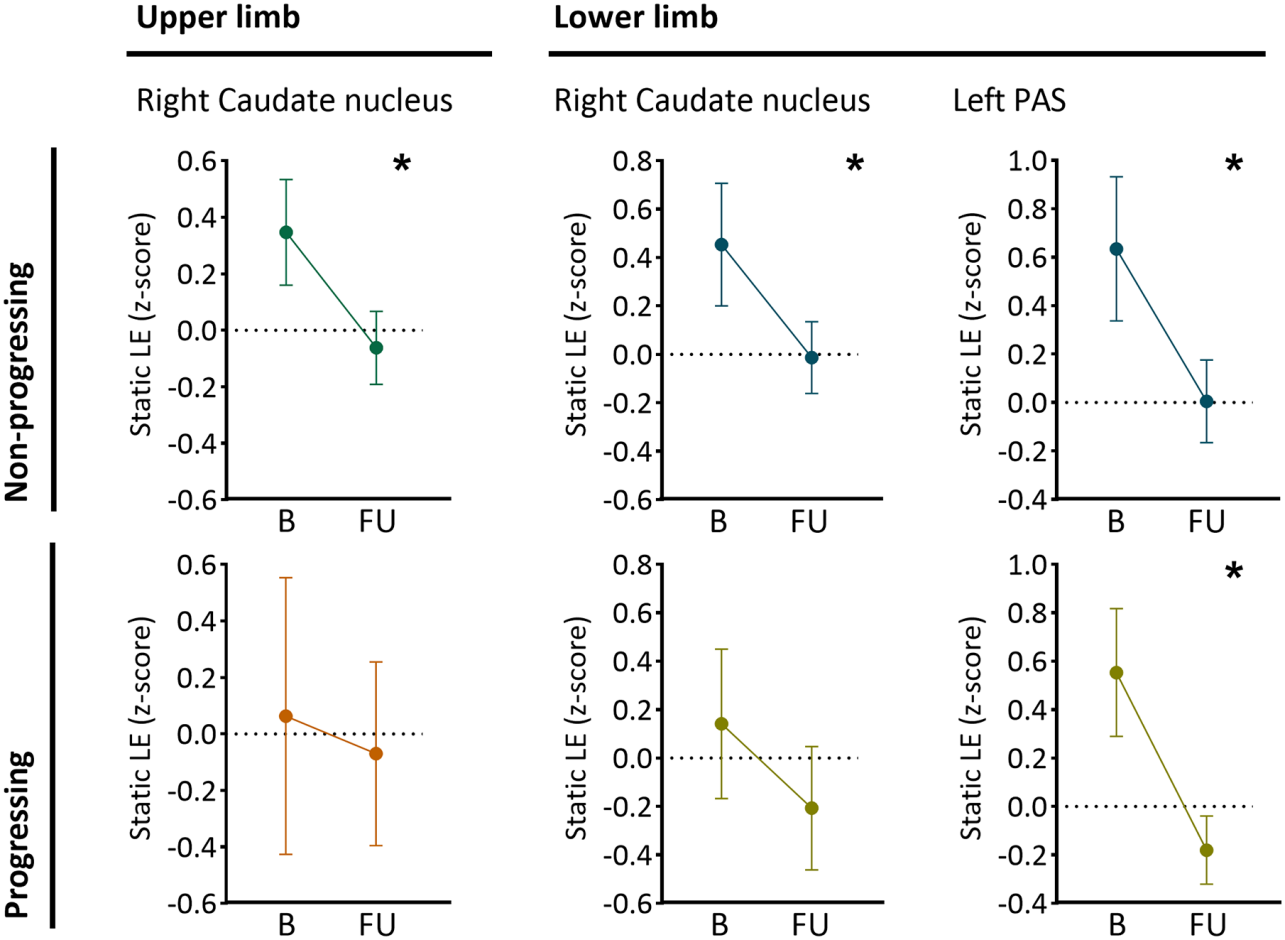
Baseline predictors of upper and lower limb disability that significantly reduced over time. The plots show the average static local efficiency (LE) at baseline (B) and follow-up (FU) for patients with and without decline in upper and lower limb functions. In non-progressing patients only, the static LE of the right caudate nucleus significantly reduced over time (upper limb *p* < 0.001 and lower limb *p* = 0.002). In both lower limb groups, a significant reduction in static LE of the left posterior associative cortex (PAS) (non-progressing and progressing; *p* < 0.001) was observed. The round symbol reflects the mean, and the error bar represents the 95% confidence interval. *Significant change over time.

Longitudinal changes of significant predictors showed unique changes (i.e. unique to one group (progressing or non-progressing)) only in static LE of the right caudate (*p* < 0.001) in upper limb non-progressing patients (Figure 3). In the lower limb groups, the static LE of the left PAS (*p* < 0.001) reduced over time in both groups and the static LE of the caudate in non-progressing patients (*p* = 0.002) (Figure 4).

## Discussion

This study investigated functional brain network changes related to upper and lower limb disability progression over 5 years in 214 people with MS, showing distinct functional network abnormalities for the upper and lower limbs. Abnormal static and dynamic network efficiency was predictive of disability progression. The SMA and caudate nucleus were predictive in both upper and lower limbs, whereas the putamen, thalamus and S1 were involved in upper limb progression only. Of these network correlates of progression, only static but not dynamic network efficiency changed over time, showing caudate reductions in non-progressing patients only.

### Baseline predictors of upper and lower limb disability in MS

Upper and lower limb progression was predicted by baseline sensorimotor network efficiency metrics, showing both shared and differential patterns. The SMA, PAS and caudate nucleus were important for both limbs, whereas the putamen, thalamus and S1 were additionally important for upper limb progression specifically. Upper limb functioning might relate more strongly to more sensory processing given that S1 and the thalamus are both important hubs in sensory processes. A central role of S1 in motor disability has been indicated by our previous cross-sectional research, that is, higher S1 efficiency in patients with severe disabilities^28^ and axonal loss in S1 tracts from early stages related to altered gait patterns and upper limb function.^29^ Besides S1, the thalamus is an expected predictor, as it is an important sensory relay centre from periphery to cortical sensorimotor areas. As such, thalamic network efficiency disturbances could have a drastic impact on information flow through the entire system. Previous research also showed higher thalamic efficiency in MS,^7^ in patients with preserved to severe disability, and increased thalamic FC^30^ and centrality^31^ in relation to cognitive symptoms. Possibly, our results could also indicate a cognitive component to upper limb progression which has been suggested before^32^ and supported by our finding that global cortical volume was only related to upper limb disability. Together our findings suggest that differential functional network mechanisms might underlie upper and lower limb disability progression in MS.

### A static and dynamic network approach

We found that abnormal static and dynamic network efficiencies were predictive of decline in upper and lower limb functions. A recent study focussing on overall disability similarly found that functional sensorimotor connectivity was predictive of clinical worsening.^8^ This and most previous studies have focussed on static functional MRI metrics, whereas we observed that abnormal baseline network dynamics were especially predictive of progression. In MS, the body of literature using dynamic connectivity and/or network approaches is still limited, and mostly focuses on cognition.^11,12,31^ Patients with cognitive impairment showed reduced FC dynamics^12^ and network centrality, in among others the sensorimotor network, hypothesized as ‘stuck’ and therefore unable to perform as needed.^11^ In this study, besides lower dynamics (possibly indicating network rigidity), we also observed a more dynamic (perhaps unstable) functional network topology predictive of progression, depending on the specific subregion. This combination warrants further study, possibly indicating that there should be a balance between flexibility and rigidity of specific regions in a normal brain network.

### Longitudinal evolution of network efficiency

Over time, we only observed a reduction in static efficiency especially of the caudate nucleus in non-progressing patients only. The caudate is part of the basal ganglia and is involved in motor processes. Reduced caudate static efficiency was observed in non-progressing patients only in both upper and lower groups, which could potentially be an early warning sign of imminent overall disability progression. Static efficiency of other regions such as the PAS declined over time in both progressing and non-progressing patients compared to controls. Together, these results suggest specific patterns in certain regions might be evident at different stages of this disease. Dynamic changes were not seen over time, only predictive at baseline, which could therefore represent a network that has started to become unstable, which might happen before clinical progression. Such a specific change was also seen in a static FC study showing increased FC in minimally disabled but decreased FC in highly disabled patients.^7^ However, it should be noted that our analyses are focused on non-directional connectivity MRI metrics with only two time points, so any causal claims remain speculative. Future studies using longer time frames and specific (and perhaps earlier) disease stages could be useful to pinpoint how static and dynamic network changes evolve and relate to disability in MS.

### Limitations

This study has several limitations to be considered. The time interval between scan sessions varied minimally between subjects, but was included as covariate. However, this window could have different effects depending on the specific disease stage, which was not further explored. Possible relapses and treatment changes during the FU period could have impacted results, which should be investigated in future work. Dynamic efficiency was calculated using a sliding window approach and not using methods without specific choices such as the Markov models. Thankfully, previous work has shown limited influence of specific variations in window length.^11^ We were unable to account for spinal cord injury, which should be investigated further in future studies. As the body of work studying network changes related to disability is small, we chose to begin with network integration and segregation, which are known crucial network features. Subsequent work should now also study additional network aspects, such as graph metrics indicating ‘importance’ of regions, such as node centrality.

## Conclusion

This study showed that dynamic functional network measures were especially predictive of limb disability progression, whereas only static network measures changed over time. Several regions including the caudate nucleus and SMA predicted progression of overall disability, whereas sensory and cognitive regions were particularly important in upper limb progression. These results therefore indicate specific mechanisms contributing to upper or lower limb impairments in MS, which should be explored further in future clinical studies.
